# Glycerol induces *G6pc* in primary mouse hepatocytes and is the preferred substrate for gluconeogenesis both *in vitro* and *in vivo*

**DOI:** 10.1074/jbc.RA119.011033

**Published:** 2019-10-23

**Authors:** Katarzyna M. Kalemba, Yujue Wang, Huiting Xu, Eric Chiles, Sara M. McMillin, Hyokjoon Kwon, Xiaoyang Su, Fredric E. Wondisford

**Affiliations:** ‡ Department of Medicine, Robert Wood Johnson Medical School, Rutgers University, New Brunswick, New Jersey 08901; § Cancer Institute of New Jersey, Rutgers University, New Brunswick, New Jersey 08903; ¶ Fred Wilson School of Pharmacy, High Point University, High Point, North Carolina

**Keywords:** gluconeogenesis, glycerol, metabolomics, hepatocyte, glucose metabolism, g6pc, pck1, glucose production, primary hepatocytes

## Abstract

Gluconeogenesis (GNG) is *de novo* production of glucose from endogenous carbon sources. Although it is a commonly studied pathway, particularly in disease, there is a lack of consensus about substrate preference. Moreover, primary hepatocytes are the current gold standard for *in vitro* liver studies, but no direct comparison of substrate preference at physiological fasting concentrations has been performed. We show that mouse primary hepatocytes prefer glycerol to pyruvate/lactate in glucose production assays and ^13^C isotope tracing studies at the high concentrations commonly used in the literature, as well as at more relevant fasting, physiological concentrations. In addition, when glycerol, pyruvate/lactate, and glutamine are all present, glycerol is responsible for over 75% of all glucose carbons labeled. We also found that glycerol can induce a rate-limiting enzyme of GNG, glucose-6-phosphatase. Lastly, we suggest that glycerol is a better substrate than pyruvate to test *in vivo* production of glucose in fasting mice. In conclusion, glycerol is the major carbon source for GNG *in vitro* and *in vivo* and should be compared with other substrates when studying GNG in the context of metabolic disease states.

## Introduction

The liver maintains euglycemia during fasting by first releasing glycogen stores and subsequently by inducing gluconeogenesis (GNG).^2^ More importantly, in diseases such as diabetes mellitus (DM), dysregulated GNG is believed to be the major cause of fasting hyperglycemia (1, 2). GNG is regulated by several hormones, including glucagon, insulin, thyroid hormone, and glucocorticoids, through changes in gluconeogenic enzyme expression, activity as well as substrate availability. During fasting, glucagon is secreted by pancreatic α-cells, binds to the glucagon receptor, and activates protein kinase A (PKA) which phosphorylates cAMP-response element–binding protein and stimulates gluconeogenic enzyme expression. The most significant enzymes are PCK1, which converts oxaloacetate to phosphoenolpyruvate, and G6PC, which converts glucose-6-phosphate to glucose (3, 4). G6PC is the terminal enzyme in GNG, and glucose-6-phosphate, regardless of how it was synthesized, must be acted upon by G6PC to become glucose. *G6pc* expression is high in the liver and renal cortex, where glucose is produced, and absent in other tissues, such as muscle and fat, where glucose is utilized (5). What remains unclear is if expression levels of these enzymes or others can explain the observed increases in hepatic glucose production in certain diseases such as DM (6, 7). Another important factor controlling gluconeogenesis is substrate availability. Biochemistry textbooks suggest that the Cori Cycle generates pyruvate and lactate from glucose metabolism in the periphery, which are then used by the liver for GNG. Lactate is rapidly oxidized to pyruvate in the liver by reducing NAD^+^ to NADH (lactate dehydrogenase), which then enters the mitochondrion and is carboxylated to oxaloacetate by pyruvate carboxylase. After reduction to malate, the four-carbon unit is transported to the cytoplasm and eventually becomes glucose. Although the malate-aspartate shuttle generates NADH from NAD^+^ in the mitochondrion, it regenerates NAD^+^ in the cytoplasm as malate is oxidized to oxaloacetate. Rapid transport of malate to the cytoplasm at the beginning of GNG is thought to limit its entry into the TCA cycle (8).

On the other hand, glycerol has a much shorter pathway to generate glucose via GNG. In fasting, glycerol derived from lipolysis of triglycerides in adipose tissue is released into the circulation and then taken up by the liver to enter the GNG (9). Hepatic glycerol kinase encoded by the X chromosome converts glycerol to glycerol-3-phosphate (G3P), which requires ATP for its phosphorylation. G3P is then oxidized to dihydroxyacetone phosphate, which enters the middle of GNG (10).

Although pyruvate and lactate have been suggested as the most important sources of endogenous glucose production, the importance of glycerol as a significant source of glucose is less clear. For example, glycerol is elevated in T2DM and predicts the worsening of hyperglycemia and insulin resistance (11–13). Livers of diet-induced obese rats also show higher rate of GNG from glycerol than from pyruvate and lactate (14), suggesting that glycerol may be a preferred substrate to pyruvate and lactate under some conditions.

Another factor that could potentially alter glucose production in primary hepatocytes is the presence of free fatty acids (FFAs). FFA metabolism results in formation of acetyl-CoA, which is a major regulator of pyruvate carboxylase (15). Whether FFAs alter GNG *in vitro* remains a question as studies have reported contradicting data (16, 17).

The majority of studies on substrate contribution to GNG were done in the 1960s (18–21). Although these were extremely thorough for the tools available at the time, new technologies have emerged that allow for a more sophisticated analysis. Thus, we used mouse primary hepatocytes to systematically determine the substrate contribution of pyruvate/lactate, glycerol, and glutamine in GNG using LC-MS measurements of ^13^C isotope–labeled metabolites. Through extensive study of primary hepatocytes, we show that glycerol is the preferred substrate for glucose production in all cases and is able to induce expression of *G6pc*, the key terminal enzyme in GNG. Our studies suggest that when investigating GNG in primary hepatocytes, substrates delivered in the most appropriate concentrations are critical to obtain the most physiologically relevant data. In addition, our *in vivo* mixed substrate tolerance test in WT mice also showed that majority of glucose carbon labeling comes from glycerol. We propose that the traditionally used pyruvate tolerance test is not be the most appropriate method for studying GNG either *in vitro* or *in vivo*.

## Results

### Primary hepatocytes produce more glucose from glycerol than from pyruvate and lactate

**Figure 1.**
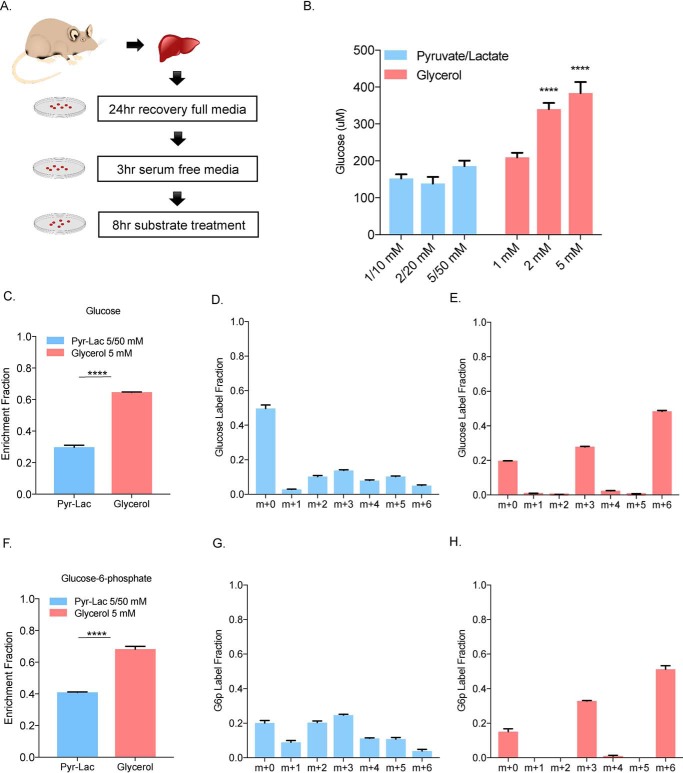
**Primary hepatocytes produce more glucose from glycerol than from pyruvate/lactate.** *A*, primary hepatocytes were isolated from 3- to 4-month-old C57B6J-albino female mice. Hepatocytes were recovered for 24 h in Williams' Media E supplemented with 10% FBS for 24 h. The following day, the cells were serum starved in Williams' Media E for 3 h. Glucose production assays were conducted over an 8-h period in glucose-free media supplemented with the indicated substrate. *B*, cells were treated with pyruvate/lactate 1:10 mixture and glycerol over a range of concentration and adjusted for basal glucose production. *C* and *F*, enrichment fraction of glucose and glucose-6-phosphate in cell extract was calculated from 5/50 mm ^13^C_3_ pyruvate/^13^C_3_ lactate and 5 mm ^13^C_3_ glycerol-treated cells. *D* and *G*, carbon label fraction pattern of glucose and glucose-6-phosphate after 5/50 mm ^13^C_3_ pyruvate/^13^C_3_ lactate treatment. *E* and *H*, 5 mm ^13^C_3_ glycerol treatment from their cell extracts.

To compare glucose production from different gluconeogenic substrates, we isolated primary hepatocytes from age-matched C57BL/6J-albino female mice fed a regular chow diet. After a 24-h recovery in complete media, the cells were serum starved for 3 h followed by a substrate challenge (Fig. 1*A*). Hepatocytes were treated with either pyruvate/lactate (1:10 molar ratio) over a range of high concentrations (1 mm pyruvate/10 mm lactate to 5 mm pyruvate/50 mm lactate) or glycerol (1 mm to 5 mm). Basal glucose production from amino acids from the media as well as glutamine were subtracted from each group based on substrate-free control group. Treatment with pyruvate and lactate in a ratio of 1:10 was employed based on physiological ratio of these substrates and their known, rapid interconversion (22). Glycerol treatment at any concentration resulted in significantly more glucose production compared with various pyruvate/lactate concentrations (Fig. 1*B*). To confirm that the increased glucose production came primarily from glycerol and not from the release of other sources such as glycogen or amino acids, we treated cells with ^13^C_3_ glycerol or ^13^C_3_ pyruvate/^13^C_3_ lactate. Based on our glucose production assays we first chose to use maximum concentrations for labeling studies of glucose production *in vitro*. We observed that glycerol treatment produced significantly higher labeled carbon enrichment fraction compared with the pyruvate/lactate treatment in both glucose (Fig. 1*C*) and glucose-6-phosphate (Fig. 1*F*), even though approximately 10 times more ^13^C_3_ was present in the pyruvate/lactate mixture. These results demonstrated that about 60% of labeled glucose carbon was derived from glycerol compared with 30% from pyruvate/lactate labeling, suggesting that glycerol is the preferred carbon source for the generation of glucose in hepatocytes at high, nonphysiological concentrations typically used in *in vitro* glucose production assays.

### ^13^C_3_ labeling shows glycerol to be a direct source of glucose

We next examined the labeling pattern of glucose using ^13^C_3_-labeled glycerol or pyruvate/lactate. ^13^C_3_-labeled pyruvate/lactate showed a variety of labeling patterns in glucose, suggesting carbon loss or exchange in other pathways such as the TCA cycle (Fig. 1*D*, *blue bars*). In contrast, ^13^C_3_ glycerol produced ∼80% labeling in glucose at m+3 and m+6 positions (Fig. 1*E*). This suggests that the three-carbon glycerol is a direct carbon source for glucose production without cycling within other metabolic pathways such as TCA cycle. Labeling of glucose-6-phosphate showed a similar pattern, with glycerol having over 80% m+3 and m+6 contribution and pyruvate/lactate a mixed distribution of labeled carbons (Fig. 1, *G* and *H*). These results suggested that glycerol is both a preferred as well as a direct substrate for GNG *in vitro* compared with pyruvate and lactate.

### Glycerol is the primary substrate for glucose production in the presence of pyruvate, lactate, and glutamine

**Figure 2.**
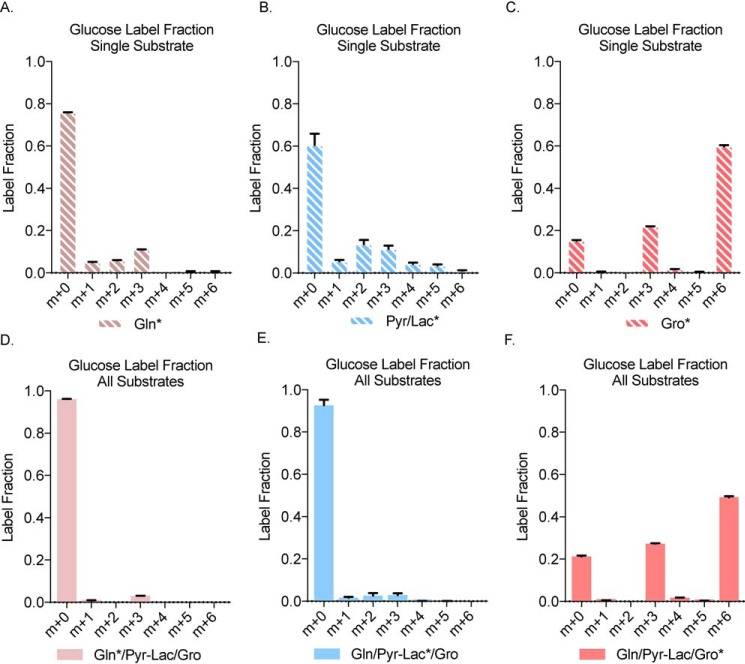
**In presence of substrates at physiological fasting concentration, glycerol is the main source of ^13^C-labeled glucose.** *A–F*, glucose label fraction from cellular extract after treatment with ^13^C_5_ glutamine alone and (*D*) ^13^C_5_ glutamine in presence of nonlabeled pyruvate/lactate and glycerol (*B*) glucose label fraction from cellular extract after treatment with ^13^C_3_ pyruvate/lactate alone and (*E*) ^13^C_3_ pyruvate/lactate in presence of nonlabeled glutamine and glycerol (*C*) glucose label fraction from cellular extract after treatment with ^13^C_3_ glycerol alone and (*F*) ^13^C_3_ glycerol in presence of nonlabeled glutamine and pyruvate/lactate. *Gln*, glutamine; *Pyr/Lac*, pyruvate/lactate; *Gro*, glycerol.

To determine whether glycerol is the preferred substrate in the context of a more physiologically relevant experiment, primary hepatocytes were treated with overnight fasting serum concentration of gluconeogenic substrates: glutamine (0.5 mm), pyruvate (0.05 mm), lactate (2.5 mm), and glycerol (0.33 mm) (23). Because glutamine is an essential component of cell culture media it was also investigated because of its potential to be a gluconeogenic substrate entering through the TCA cycle. We first characterized individually labeled substrates (glutamine, pyruvate/lactate, or glycerol) at physiological fasting concentrations (Fig. 2, *A–C*). Glutamine and pyruvate/lactate showed a variety of glucose carbon labeling (Fig. 2, *A* and *B*), whereas glycerol produced a clear m+3 and m+6 carbon pattern in glucose labeling (Fig. 2*C*) as previously observed in the higher concentration treatments (Fig. 1*E*).

We next treated primary hepatocytes with fasting serum physiological concentrations of all substrates, mimicking the presence of all substrates *in vivo* (Fig. 2, *D–F*). In the first group, we combined ^13^C_5_-labeled glutamine and nonlabeled pyruvate/lactate and glycerol (Fig. 2*D*). The other two groups had ^13^C_3_-labeled pyruvate/lactate (Fig. 2*E*) or ^13^C_3_-labeled glycerol along with other nonlabeled substrates (Fig. 2*F*). We observed that only about 10% of glucose was labeled by ^13^C_5_ glutamine (Fig. 2*D*) or ^13^C_3_-labeled pyruvate/lactate (Fig. 2*E*) when all substrates are combined at physiological concentrations. Most interestingly, however, when hepatocytes were treated with ^13^C_3_ glycerol along with other nonlabeled substrates, about 80% of glucose was labeled, suggesting that glycerol is the dominant substrate in glucose production in hepatocytes, even in the presence of all known substrates (Fig. 2*F*). The carbon-labeling pattern also showed the same m+3 and m+6 pattern reflecting incorporation of either one or two intact, labeled ^13^C_3_ glycerol molecules.

### Glycerol is directly incorporated into glucose whereas pyruvate and lactate first circulate in the TCA cycle

**Figure 3.**
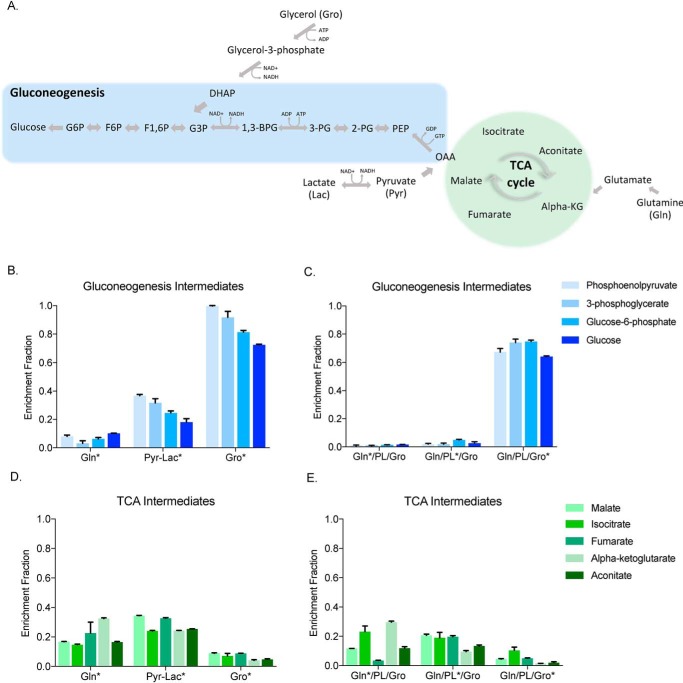
**Glycerol carbon mainly produces gluconeogenic intermediates and enters the TCA cycle to a minimal extent.** *A*, simplified overview of substrate entry into gluconeogenesis and TCA cycle. *B–E*, gluconeogenic intermediates (phosphoenolpyruvate, 3-phosphoglycerate, glucose-6-phosphate and glucose) and (*D*) TCA intermediates (malate, isocitrate, fumarate, α-ketoglutarate, aconitate) cellular extract enrichment fraction from single ^13^C-labeled substrates at their physiological concentration (*C*) gluconeogenic and (*E*) TCA intermediates cellular extract enrichment fraction of all substrate groups with first ^13^C_5_ glutamine in presence of unlabeled pyruvate/lactate and glycerol, then ^13^C_3_ pyruvate/lactate in presence of unlabeled glutamine and glycerol and finally ^13^C_3_ glycerol in presence of unlabeled glutamine and pyruvate/lactate.

To investigate further the metabolic fates of each substrate, we used single-labeled substrates (Fig. 3, *B* and *D*) as well as combination groups with individually labeled substrates (Fig. 3, *C* and *E*). We tracked the metabolic fate of the labeled substrates in GNG and the TCA cycle by looking at various intermediates as shown (Fig. 3*A*). When examining the GNG intermediates phosphoenolpyruvate, 3-phosphoglycerate, and glucose-6-phosphate and glucose, we found that ^13^C_3_ glycerol alone contributed on average ∼80% of labeled carbon to GNG intermediates and glucose (Fig. 3*B*). In the presence of three substrates, glycerol was also predominately used in GNG contributing over 70% of labeled carbon to each of these intermediates and glucose (Fig. 3*C*). ^13^C_5_ glutamine and ^13^C_3_ pyruvate/lactate, in contrast, labeled intermediates and glucose at a much lower level, especially in the context of all three substrates (Fig. 3, *B* and *C*). Our second question focused on the amount of labeled carbon entering the TCA cycle as reflected in labeling of malate, citrate, fumarate, α-ketoglutarate and aconitate. We observed that only ^13^C_5_ glutamine and ^13^C_3_ pyruvate/lactate carbons contributed significantly to labeling TCA cycle intermediates (Fig. 3, *D* and *E*). As expected, the high labeling was derived from ^13^C_5_ glutamine because of its entry into the TCA cycle directly through α-ketoglutarate (Fig. 3, *D* and *E*). Somewhat unexpectedly, ^13^C_3_ pyruvate/lactate also significantly labeled TCA intermediates. These data suggest that glycerol is a preferred substrate for GNG and directly contributes to glucose production without generating TCA cycle intermediates.

### Presence of FFAs does not alter glycerol's contribution to glucose carbon

**Figure 4.**
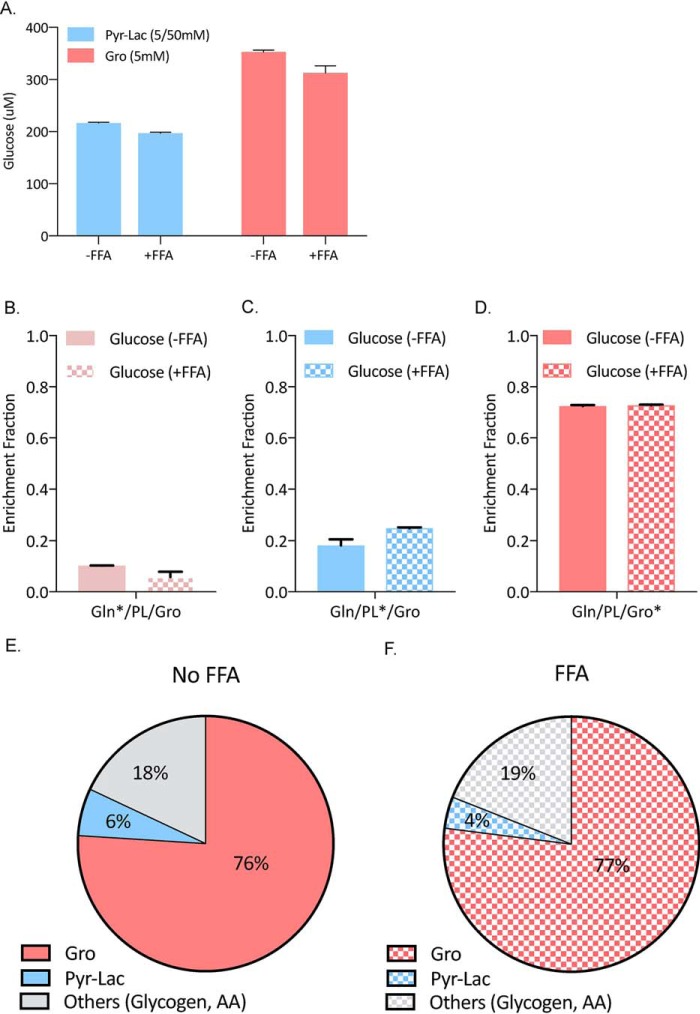
**Presence of free fatty acids does not change the substrate preference for glycerol.** *A*, glucose production with high concentrations of glycerol (5 mm) and pyruvate/lactate (5/50 mm) in presence or absence of 200 μm palmitate and 200 μm oleate conjugated to BSA. *B–D*, glucose enrichment fraction from physiological fasting concentrations of substrates with or without palmitate (200 μm) and oleate (200 μm) conjugated to BSA. *B*, ^13^C_5_ glutamine in presence of unlabeled pyruvate/lactate and glycerol. *C*, ^13^C_3_ pyruvate/lactate in presence of unlabeled glutamine and glycerol. *D*, ^13^C_3_ glycerol in presence of unlabeled glutamine and pyruvate/lactate. *E* and *F*, final glucose carbon contributions determined using the mathematical model without (*E*) and with (*F*) FFA supplementation.

Based on glucose production assays seen in Fig. 1*A*, we treated primary hepatocytes with high concentrations of pyruvate/lactate (5/50 mm) and glycerol (5 mm) with or without palmitate and oleate. No significant differences in glucose production were observed after the addition of FFA (Fig. 4*A*). Next, using the same experimental conditions as in Fig. 3, we treated mouse primary hepatocytes with labeled substrate mixtures in presence or absence of a mixture of 200 μm palmitate and 200 μm oleate conjugated to BSA (Fig. 4, *B–D*). We chose these concentrations because FFA fasting serum concentrations range between 300 and 600 μm (24–28). We also observed no significant differences in labeling fractions between these two groups. Because FFAs were dissolved in ethanol prior to application to primary cultures, we also determined the NADH/NAD^+^ and DHAP/G3P ratios, which could be artificially altered by the presence of ethanol. No significant changes were observed in either ratios, indicating that the residual ethanol in the tissue culture medium did not affect basic energy pathways in the primary hepatocyte cultures (Fig. S1).

Next, we developed and validated a mathematical model for primary mouse hepatocyte glucose production in the presence of fasting concentrations of glycerol, pyruvate/lactate, and glutamine (Fig. S3). Based on this model, we calculated the contribution of each substrate to final glucose carbons. In presence of all GNG substrates, 76% of glucose label was derived from glycerol, 18% was from other substrates such as glycogen and amino acids present in the medium, and only 6% was derived from pyruvate/lactate (Fig. 4*E*). The presence of FFAs did not significantly alter these percentages (Fig. 4*F*).

To validate this method, we first compared the simulated and observed labeling patterns of glucose, glucose-6-phosphate, phosphoenolpyruvate, and pyruvate when ^13^C_3_ lactate or glycerol was used as tracer (Fig. S4, *A* and *B*). The simulated and observed patterns are consistent, suggesting a good estimation of the fluxes based on this model. Because all the fluxes are calculated using ^13^C_3_ glycerol and ^13^C_3_ lactate data, it is important to know whether these fluxes also predict the labeling patterns from other tracers. Therefore, we simulated the labeling patterns under ^13^C_5_ glutamine tracer and found that simulated and observed patterns are consistent (Fig. S4*C*). Overall, these data suggest our method can be used to accurately estimate GNG fluxes in primary hepatocytes cultured with physiological concentration of substrates.

### Glycerol induces G6pc and reduces Pck1 expression in primary hepatocytes

**Figure 5.**
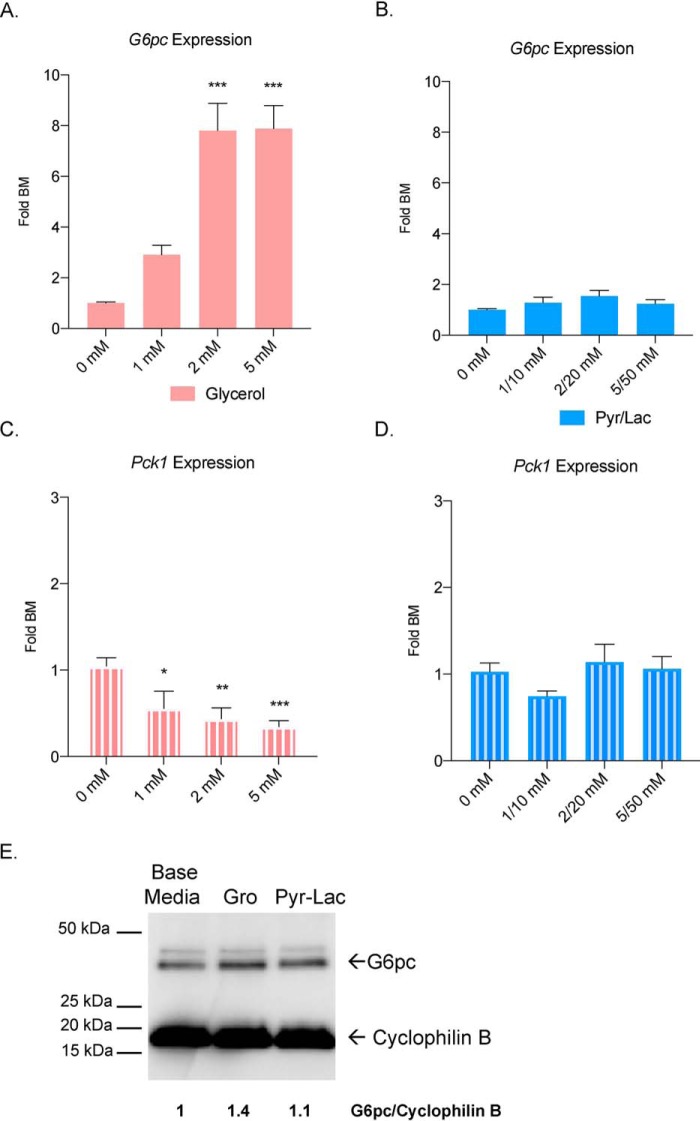
**Glycerol induces *G6pc* and suppresses *Pck1* expression in primary mouse hepatocytes.** *A* and *B*, *G6pc* mRNA expression after glycerol fold substrate free base media (*BM*) and (*B*) pyruvate/lactate treatment over increasing concentrations. *C* and *D*, *Pck1* mRNA expression after (*C*) glycerol and (*D*) pyruvate/lactate treatment over increasing concentrations. *E*, Western blotting of G6pc protein in glycerol- (5 mm) and pyruvate/lactate– (5/50 mm) treated samples. Values (arbitrary units) indicate expression of G6pc normalized to cyclophilin B.

To investigate why glycerol is a preferred substrate in GNG, we measured gene expression of key gluconeogenic enzymes (*G6pc* and *Pck1*) after glycerol and pyruvate/lactate treatment using RT-qPCR. A significant induction of *G6pc* expression, the terminal enzyme in GNG, was observed after an 8-h glycerol treatment in mouse primary hepatocytes over a range of concentrations (Fig. 5*A*), plateauing around 2 mm. There was no significant change in gene expression after pyruvate/lactate treatment compared with control base media (Fig. 5*B*). We also confirmed the protein expression for G6pc via Western Blotting. A 1.4 fold-change was observed in the glycerol stimulated group compared to base media (Fig. 5*E*). Next, we examined expression of *Pck1*, an enzyme in the early part of GNG that catalyzes the conversion of oxaloacetate to phosphoenolpyruvate and is required for glucose production from pyruvate/lactate. Interestingly, glycerol suppressed the expression of *Pck1* (Fig. 5*C*), whereas pyruvate/lactate treatment did not alter *Pck1* expression compared with the control hepatocytes (Fig. 5*D*). We also tested whether hormones such as insulin and glucagon would affect gene expression. Insulin significantly reduced *G6pc* and *Pck1* expression in pyruvate/lactate–treated hepatocytes (Fig. S2, *A* and *B*). In the glycerol group, insulin only significantly inhibited *G6pc* expression (Fig. S2, *A* and *B*). Glucagon on the other hand, stimulated *G6pc* and *Pck1* similarly in the base media and pyruvate/lactate groups. Glycerol had a much larger increase in *G6pc* expression and much smaller response to *Pck1* compared with the other groups (Fig. S2, *C* and *D*).

### Glycerol becomes the dominant substrate for glucose production in WT mice

**Figure 6.**
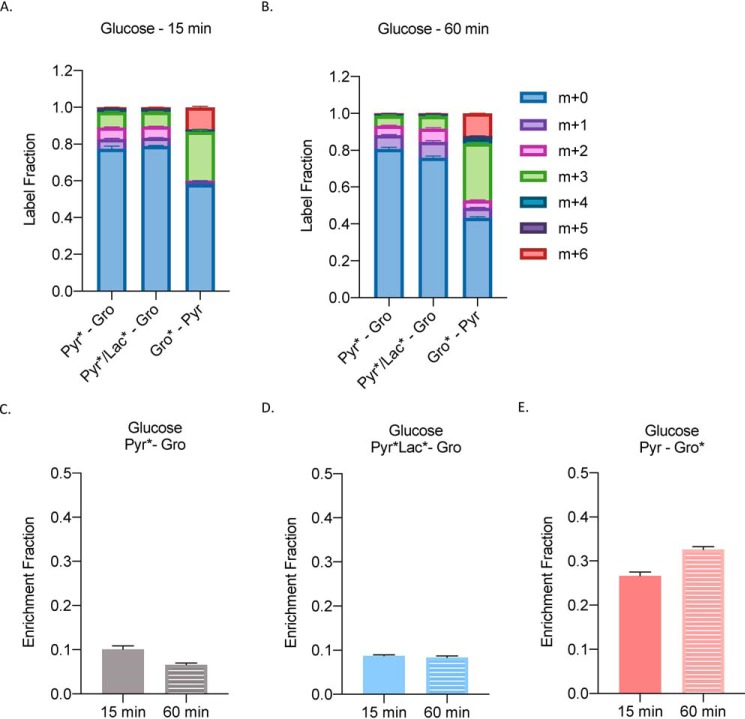
**Glycerol becomes the dominant substrate for glucose production in WT mice.** *A* and *B*, label fraction of glucose (*A*) 15 min and (*B*) 60 min after injection of a mixture of substrates in the following groups: 1 mm ^13^C_3_ pyruvate–1 mm glycerol; 0.1 mm/0.9 mm ^13^C_3_ pyruvate/^13^C_3_ lactate–1 mm glycerol; and 1 mm pyruvate–1 mm ^13^C_3_ glycerol. *C–E*, enrichment fraction of glucose in the same groups. *n* = 3 per group of overnight fasted WT males.

Next, we wanted to study how glycerol would label glucose *in vivo* after a bolus injection of a substrate mixture. Similar to our experiment in Fig. 3, we set up the following combination groups: 1) 1 mm ^13^C_3_ pyruvate–1 mm glycerol; 2) 0.1 mm/0.9 mm ^13^C_3_ pyruvate/^13^C_3_ lactate–1 mm glycerol, and 3) 1 mm pyruvate–1 mm ^13^C_3_ glycerol. Traditionally, a pyruvate tolerance test is done at a 1 mm concentration; therefore, we wanted to keep the substrates in that range and observe which would dominate in a glucose production assay. Fifteen min post injection (Fig. 6*A*), we observed a similar pattern of label fractions as seen in Fig. 3. Pyruvate and pyruvate/lactate mixture groups showed a range of the labeled carbon whereas glycerol primarily showed a m+3 and m+6 label. At 60 min (Fig. 6*B*), pyruvate and pyruvate/lactate mixture groups stayed consistent, whereas glycerol's carbon label increased and other labeling patterns were observed. Next, we looked at the enrichment fraction of glucose from these groups. We found that pyruvate and pyruvate/lactate groups labeled only about 10% of glucose at both time points (Fig. 6, *C* and *D*). Glycerol, however, labeled almost 30% of glucose at 15 min and slightly more at 60 min (Fig. 6*E*).

## Discussion

It is conventionally accepted that pyruvate and lactate are the main substrates for GNG, and these substrates are extensively used to test hepatic glucose production both *in vitro* and *in vivo* and are employed generally at supraphysiological concentrations (29–35). There are no studies, however, showing glucose production using physiological fasting concentrations of substrates or studies using a combination of substrates. In this study we show: 1) glycerol is the main source of glucose carbon by itself as well as in the presence of other GNG substrates, 2) pyruvate and lactate contribute to TCA cycle intermediates, 3) the presence of FFAs in media does not alter glycerol's glucose contribution, and 4) glycerol but not pyruvate/lactate induces expression of G6PC and inhibits expression of PCK1.

Early studies suggested that pyruvate/lactate was the main substrate for glucose production in the liver (36–38). Based on these and other observations, *in vitro* and *in vivo* pyruvate tests have been used to measure hepatic glucose production (3, 39, 40). However, our analysis shows that glycerol alone, or in combination with other major substrates, accounts for the majority of the labeled glucose carbon in mouse primary hepatocytes. We suggest that glycerol is a more powerful substrate than previously estimated. Previous work also supports a critical role for glycerol. For example, elevated serum glycerol is found in obese T2DM patients and predicts the development of hyperglycemia (11, 25). Furthermore, glucose production from glycerol in Type 2 DM subjects displays a 1.7-fold increase compared with normal subjects (41); and glycerol incorporation into glucose was reported to have a 3-fold increase in Type 2 DM compared with controls (27).

Pyruvate enters GNG after conversion to oxaloacetate in the mitochondrion, which is then transported to the cytosol for glucose production via the malate shuttle (4, 42, 43). However, our studies show similar labeling of TCA intermediates to that observed from glutamine, a substrate that must label TCA intermediates. Our findings suggest that a significant amount of pyruvate in primary hepatocytes enters the TCA cycle prior to GNG and this event could limit glucose production.

A factor normally absent in cell culture media that could potentially alter substrate preference for glucose is FFA. Acetyl-CoA is produced from β-oxidation of fatty acids and allosterically activates pyruvate carboxylase, which then promotes the entry of pyruvate into the GNG pathway (15). For this reason, we added oleic and palmitic acid conjugated to BSA to primary hepatocyte media, and similar to Fig. 3, we measured the contribution of each GNG substrate to glucose carbons. The addition of FFA did not increase glucose production or shift substrate preference in primary hepatocytes, confirming that hepatocytes prefer glycerol even in the presence of FFA. A major concern with using FFAs in culture media is the presence of ethanol solvent, which when metabolized could alter NADH/NAD^+^ ratios. We determined the NADH/NAD^+^ and DHAP/G3P ratio but did not observe any differences after FFA treatment (Fig. S1).

Fatty acids are said to play a role in the development of insulin resistance in muscle and liver (44), but the role of FFAs on hepatic glucose production remains unclear. Collins *et al.* (16) reported that oleate in concentrations ranging from 100 μm to 1.5 mm induces glucose production in primary hepatocytes. On the other hand, a different study reported that oleate and palmitate attenuated GNG in primary hepatocytes at 1 mm (45). One of the hypotheses for glucose production involves generation of succinate from free acetate produced by FFAs (17). Although we observed elevated succinate levels in FFA-treated hepatocytes (data not shown), this elevation did not result in any alteration in glucose carbon preference.

Another finding in this study is that glycerol metabolism increases *G6pc* expression. Previously, glycerol was shown to slightly increase levels of *G6pc* in a rat hepatoma cell line (46); however, an effect in primary hepatocytes has not been reported. The importance of elevated *G6pc* mRNA levels in mediating hyperglycemia, however, is still unclear. Clore *et al.* (25) reported that in morbidly obese patients with T2DM, G6PC activity was significantly increased compared with controls. The same phenomenon was observed in diabetic rats (47). In another study, increased *G6pc* activity was correlated with hyperglycemia (48). On the other hand, other work showed that *G6PC* expression is not elevated in patients with T2DM (6). A minimal glycerol concentration required for *G6PC* induction and acute *versus* chronic exposure to glycerol could be other reasons why data are conflicting in patients with T2DM. In this study, we observed an ∼8-fold, concentration-dependent increase in *G6pc* expression (Fig. 5) in primary mouse hepatocytes, which correlated with the marked increase in glucose production we observed (Fig. 1).

In contrast to induction of *G6pc* expression, *Pck1* expression was inhibited by glycerol treatment. In fact, primary hepatocytes treated with glycerol showed a strong, concentration-dependent inhibition in *Pck1* expression. In obese humans, serum and liver glycerol are significantly increased (11) and *PCK1* expression is lower compared with controls (6). Intriguingly, it has been shown that mice with a liver-specific knockout of *Pck1* develop hepatic steatosis (49), whereas *Pck1* overexpression in skeletal muscle reduced weight gain and had a positive effect on metabolism and energy in mice (50). Hepatic steatosis is found in as many as 75% of individuals with T2DM (51), and our studies suggest that glycerol inhibition of *Pck1* might contribute to the development of fatty liver.

The activation of gluconeogenesis often results from glucagon signaling. We observed that in hepatocytes treated with both glucagon and glycerol, *G6pc* expression increased even further compared with basal and pyruvate/lactate media (Fig. S2*C*). Consistent with these results, T2DM patients have fasting hyperglycemia because of inappropriate glucagon secretion and increased GNG (2, 52). Interestingly, *Pck1* expression was lower in the glycerol group compared with basal and pyruvate/lactate media. Although the mechanism of glycerol's activation of *G6pc* and suppression of *Pck1* remains unanswered, these data provide a direction for future mechanistic investigations.

Although our model primarily investigates the role of glycerol in primary hepatocytes, we also observed that it applies to *in vivo* physiology. In Fig. 6, we investigated substrate utilization in GNG in overnight WT fasted mice. We tried to mimic a traditional pyruvate tolerance test (PTT) but also used a combination of substrates to observe which one would dominate (similar to our *in vitro* experiment from Fig. 3). It is clear from these studies that glycerol is the preferred *in vivo* substrate. A PTT is commonly used to assess gluconeogenic capacity in a number of mouse models (39). Our critical finding suggests that compared with a traditionally PTT, glycerol might be a better substrate for evaluating GNG in mice.

The primary hepatocyte system allows an investigation of GNG without the contribution of the Cori cycle or the influence of serum hormones. Although there is an advantage to studying pathways with this approach, it is of course limited. Our findings are meant to better approximate but not recapitulate *in vivo* metabolism. However, because of the feasibility and popularity of using primary hepatocyte cultures as a model system, we suggest that it is critical to the field to provide a more physiologically valid *in vitro* model. We believe our findings provide such a model.

We report a previously underestimated importance of glycerol in glucose production in primary mouse hepatocytes as well in *in vivo* glucose production assays. As a substrate for GNG, glycerol is capable of producing more glucose than other known substrates. It also induces the terminal enzymatic step in the GNG pathway (*G6pc*) and inhibits an early GNG enzyme (*Pck1*) that is critical for entry of pyruvate and substrates metabolized to pyruvate into GNG. We believe that glycerol's importance in GNG may be underappreciated, especially in the context of current *in vitro* and *in vivo* assays used to measure hepatic glucose production.

## Experimental procedures

### Animal experiments

All animal protocols were approved by the Institutional Animal Care and Use Committee of Rutgers University. B6(Cg)-Tyrc-2j/J or B6 albino mice were obtained from The Jackson Laboratory (Bar Harbor, ME). Mice were fed *ad libitum* standard chow and kept at 12 h light/12 h dark cycles.

### Primary hepatocyte isolation

Age-matched C57/B6J albino females between 3 and 4 months were used for primary culture isolation. Mice were first anesthetized using a ketamine/xylazine mixture (Henry Schein Inc.). To obtain primary hepatocytes, the hepatic portal vein was cannulated and perfused with Krebs-Ringer solution containing EGTA for 10 min at 37 °C. After the first wash, a second Krebs-Ringer wash containing CaCl and Liberase^TM^ (Roche) was used for 10 min at 37 °C. Hepatocytes were filtered through a gauze mesh and resuspended in plating media: Williams' Media E (Sigma);10% FBS (Sigma); 200 nm dexamethasone (Sigma); 5 ml penicillin/streptomycin (Fisher); and 2 mm l-glutamine (Fisher). Cells were plated at a density of 0.3 × 10^6^ on 6-well collagen-coated (Sigma) plate. Hepatocytes were allowed to recover overnight and experiments were started 24 h post isolation.

### Glucose production assays

Prior to each glucose production assay, cells were serum starved for 3 h. The assay was done in basal media, DMEM (Gibco) containing no glucose or pyruvate but supplemented with l-glutamine (2 mm) unless otherwise stated, HEPES (1.76 g), and penicillin/streptomycin (5 ml). For substrate glucose production assays, basal media were supplemented with l-glutamine, glycerol, or a sodium pyruvate/lactate mixture. For labeled experiments, the same conditions were used but uniformly labeled stable isotope substrates were obtained from Cambridge Isotopes: ^13^C-sodium pyruvate (CLM-2440), ^13^C-sodium lactate (CLM-10768), ^13^C-l-glutamine (CLM-1822), and ^13^C-glycerol (CLM-1510). Free fatty acids were dissolved in ethanol and then conjugated with 2% BSA (0.3 mm). Glucose measurements were done enzymatically with Glucose Assay Kit (Abcam).

### Real time qRT-PCR analysis

Total RNA was isolated from primary hepatocytes and mouse livers using TRIzol method. cDNA was obtained using iScript (Bio-Rad) and then subjected to qRT-PCR analysis using SYBR Green (Bio-Rad) according to manufacturer's protocol. The primers used for the analysis were the following: *G6pc*, forward 5′-CAGCAAGGTAGATCCGGGA-3″, reverse 5′-AAAAAGCCAACGTATGGATTCCG-3′; *Pck1*, forward 5′-AGCATTCAACGCCAGGTTC-3″, reverse 5′-CGAGTCTGTCAGTTCAATACCAA-3′; *Actb*, forward 5′-CCAGTTGGTAACAATGCCATG-3″, reverse 5′-GGCTGTATTCCCCTCCATCG-3′.

### Western blotting

Hepatocytes were lysed in Laemmli buffer with β-mercaptoethanol. Eight μl of cell lysate was run by a Bio-Rad Western blotting protocol. G6pc antibody was generously provided by Dr. Fabienne Rajas. Cyclophilin B was used as a loading control. Protein expression was normalized to its loading control and subsequently to -fold change of Base Media control. Densitometric analysis was performed on ImageLab software (Bio-Rad).

### Metabolite extraction for LC-MS

Fresh primary hepatocytes were treated with 40:40:20 methanol:acetonitrile:water solution with 0.1% formic acid, followed by incubation on ice for 5 min, and neutralized by NH_4_HCO^3^ addition. Then they were centrifuged at 4 °C and 16,000 × *g* for 10 min. The supernatant was transferred to a clean tube and stored at −80 °C until analysis.

### Metabolite isotope tracing

Conditions were optimized on an HPLC-ESI-MS system fitted with a Dionex UltiMate 3000 HPLC and a Thermo Scientific Q Exactive Plus MS. The HPLC was fitted with a Waters XBridge BEH Amide column (2.1 mm × 150 mm, 2.5 μm particle size, 130 Å pore size) coupled with a Waters XBridge BEH XP VanGuard cartridge (2.1 mm x 5 mm, 2.5 μm particle size, 130 Å pore size) guard column. The column over temperature was set to 25 °C. The solvent A consisted of water/acetonitrile (95:5, v/v) with 20 mm NH_3_AC and 20 mm NH_3_OH at pH 9. The solvent B consisted of acetonitrile/water (80:20, v/v) with 20 mm NH_3_AC and 20 mm NH_3_OH at pH 9 in the following solvent B percentages over time: 0 min, 100%; 3 min, 100%; 3.2 min, 90%; 6.2 min, 90%; 6.5 min, 80%; 10.5 min, 80%; 10.7 min, 70%; 13.5 min, 70%; 13.7 min, 45%; 16 min, 45%; 16.5 min, 100%. The flow rate was set to 300 μl/min with an injection volume 5 μl. The column temperature was set at 25 °C. MS scans were obtained in negative ion mode with a resolution of 70,000 at *m*/*z* 200, in addition to an automatic gain control target of 3 × 10^6^ and *m*/*z* scan range of 72 to 1000. Metabolite data were obtained using the MAVEN software package (56) with each labeled isotope fraction (mass accuracy window 5 ppm). The isotope natural abundance and tracer isotopic impurity were corrected using AccuCor (57).

### Quantification of gluconeogenic fluxes in primary hepatocytes

The gluconeogenic fluxes are calculated by elementary metabolite unit–based methods (53–55). In brief, a simplified flux network was constructed and is described in the supporting data.

### Bolus injection study

Age-matched male mice were fasted overnight and immunoprecipitate injected with the mixture of substrates in the following groups: 1 mm ^13^C_3_ pyruvate–1 mm glycerol; 0.1 mm/0.9 mm ^13^C_3_ pyruvate/^13^C_3_ lactate–1 mm glycerol; and 1 mm pyruvate–1 mm ^13^C_3_ glycerol. Blood was sampled at 15 and 60 min and results were analyzed on the LC-MS.

### Statistics and analysis

All analysis and graphs were done on GraphPad Prism software. Statistics involved a Student's *t* test or analysis of variance (ANOVA) as appropriate.

## Author contributions

K. M. K., Y. W., H. X., E. C., and S. M. M. investigation; K. M. K., H. X., and X. S. methodology; K. M. K. writing-original draft; K. M. K., Y. W., E. C., S. M. M., H. K., X. S., and F. E. W. writing-review and editing; Y. W. validation; H. X. data curation; H. X., E. C., and S. M. M. formal analysis; H. K., X. S., and F. E. W. supervision; F. E. W. funding acquisition.

## Supplementary Material

Supporting Information

## Footnotes

2 The abbreviations used are:

GNG: gluconeogenesis
DM: diabetes mellitus
TCA: tricarboxylic acid
G3P: glycerol-3-phosphate
T2DM: Type 2 DM
PTT: pyruvate tolerance test.
