# Supplementary material for: Glycerol induces G6pc in primary mouse hepatocytes and is the preferred substrate for gluconeogenesis both in vitro and in vivo
Source: J Biol Chem. 2019 Oct 23;294(48):18017–28. doi: 10.1074/jbc.RA119.011033 (PMC6885632; doi:10.1074/jbc.RA119.011033)
Supplement: Supporting Information [file supp_294_48_18017__index.html]

Glycerol induces G6pc in primary mouse hepatocytes and is the preferred substrate for gluconeogenesis both in vitro and in vivo — EDITORS' PICK: Glycerol induces G6PC and is the preferred substrate — Supporting Information 

# Glycerol induces *G6pc* in primary mouse hepatocytes and is the preferred substrate for gluconeogenesis both *in vitro* and *in vivo*

## Supporting Information

- Supporting Information (to be published online) - More detail on metabolomic studies
