# Supplementary material for: Glycerol induces G6pc in primary mouse hepatocytes and is the preferred substrate for gluconeogenesis both in vitro and in vivo
Source: J Biol Chem. 2019 Oct 23;294(48):18017–28. doi: 10.1074/jbc.RA119.011033 (PMC6885632; doi:10.1074/jbc.RA119.011033)
Supplement: Supporting Information [file supp_RA119.011033_155785_1_supp_411417_pzdpdp.pdf]

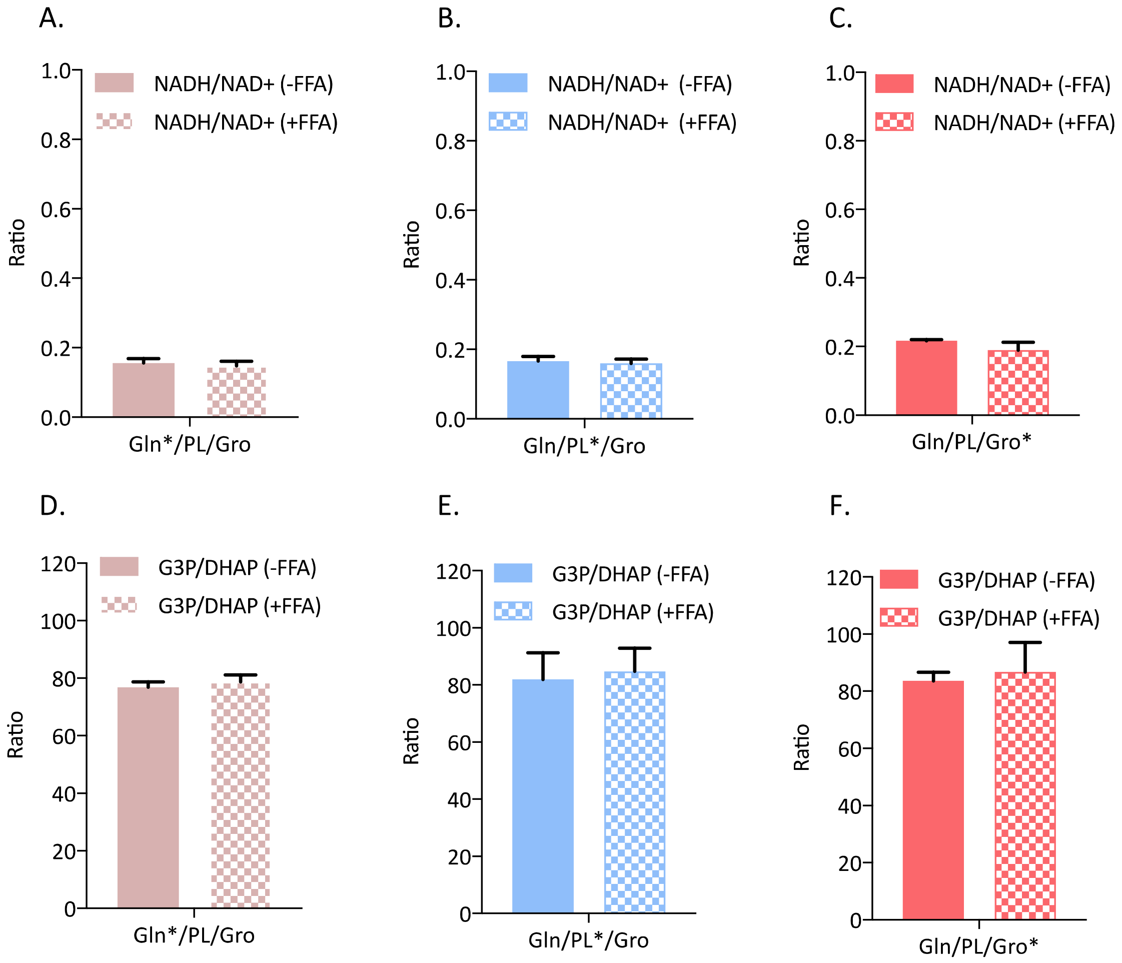

Figure S1: Energy ratios remain unchanged in the presence of ethanol in the media

$NADH/NAD^+$ ratios in primary hepatocyte cultures treated with physiological concentrations of substrates: **(a)** $^{13}C_5$ glutamine in presence of unlabeled pyruvate/lactate and glycerol, **(b)** $^{13}C_3$ pyruvate/lactate in presence of unlabeled glutamine and glycerol and **(c)** $^{13}C_3$ glycerol in presence of unlabeled glutamine and pyruvate/lactate with or without palmitate (200 µM) and oleate (200 µM) conjugated to BSA DHAP/G3P ratios in primary hepatocyte cultures treated with physiological concentrations of substrates: **(d)** $^{13}C_5$ glutamine in presence of unlabeled pyruvate/lactate and glycerol, **(e)** $^{13}C_3$ pyruvate/lactate in presence of unlabeled glutamine and glycerol, and **(f)** $^{13}C_3$ glycerol in presence of unlabeled glutamine and pyruvate/lactate with or without palmitate (200 µM) and oleate (200 µM) conjugated to BSA

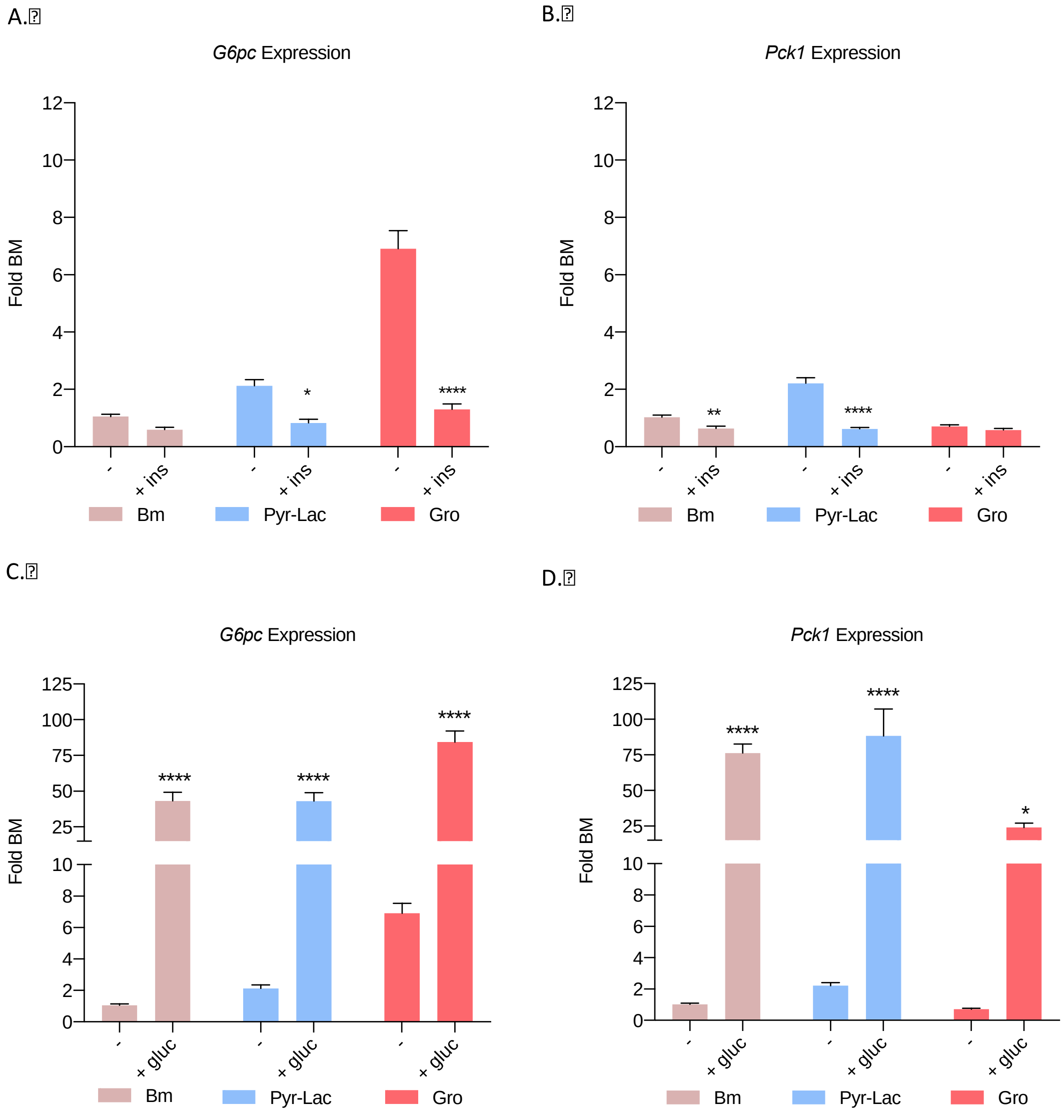

Figure S2: Insulin suppresses and glucagon induces gluconeogenic genes

(a) *G6pc* and **(b)** *Pck1* expression after 8 hour treatment of primary hepatocytes with control base media, 5/50 mM pyruvate and 5 mM glycerol groups with 1nM insulin **(c)** *G6pc* and **(d)** *Pck1* expression after 8 hour treatment of primary hepatocytes with control base media, 5/50 mM pyruvate and 5 mM glycerol groups with 10nM glucagon

Figure S3: Quantification of gluconeogenic fluxes in primary hepatocytes

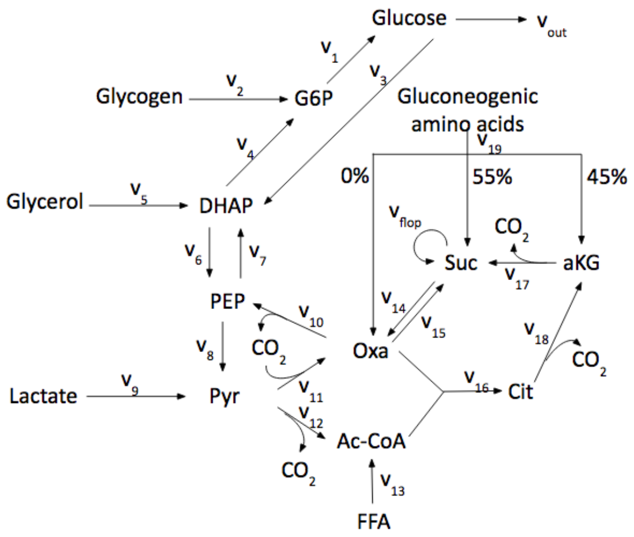

The gluconeogenic fluxes are calculated by an elementary metabolite units (EMU) based method (53). In brief, a simplified flux network is constructed as shown below:

V_2, V_5, V_9, V_{13} and V_{19} are input fluxes from glycogen, glycerol, lactate, free fatty acid (FFA) and gluconeogenic amino acids respectively. V_{11} is pyruvate carboxylase flux that incorporates carbon dioxide. V_{10}, V_{12}, V_{17} and V_{18} are decarboxylase fluxes that release carbon dioxide. V_{flop} is an infinitely large flux to account for the symmetry of succinate. V_{out} is the output flux which represents the release of glucose into the media from hepatocytes. Therefore, the absolute value of V_{out} can be measured as the glucose production rate in the media.

The contribution of gluconeogenic amino acids consists of three components: 1. via oxaloacetate (Asp, Asn); 2. via fumarate or succinyl CoA (Phe, Tyr, Ile, Met, Val, Thr); 3. via a-ketoglutarate (Gln, Glu, Pro, His, Arg). The media used in the experiment contains 0.80 mM Ile, 0.80 mM Val, 0.80 mM Thr and 2.00 mM Gln. Therefore, the total substrate concentration in the three possible pathways are 0, 2.40 and 2.00 mM respectively. Assuming proportional contribution by the three routes, the three sub-fluxes to oxaloacetate, succinate and alpha-ketoglutarate are 0, 55% and 45% of V_{19} respectively.

The metabolite mass balance leads to the following equations:

Glucose:	$V_1 - 0.5*V_3 = V_{out}$	
G6P:	$V_1 - V_4 = V_2$	
DHAP:	$2*V_4 + V_6 - V_3 - V_7 = V_5$	
PEP:	$V_7 + V_8 - V_6 - V_{10} = 0$	
Pyr:	$V_{11} + V_{12} - V_8 = V_9$	[S1]
Oxa:	$V_{16} + V_{10} + V_{15} - V_{11} - V_{14} = 0.18*V_{19}$	
Ac-CoA:	$V_{16} - V_{12} = V_{13}$	
Suc:	$V_{14} - V_{15} - V_{17} = 0.45*V_{19}$	
Cit:	$V_{16} - V_{18} = 0$	
aKG:	$V_{17} - V_{18} = 0.37*V_{19}$	

Balance of input and output mass leads to the following equation:

$$6*V_{out} = 6*V_2 + 3*V_5 + 3*V_9 + 2*V_{13} + 4.37*V_{19} - V_{12} - V_{10} - V_{17} - V_{18} + V_{11} \quad [S2]$$

Nine fluxes are designated free fluxes: V_2, V_3, V_5, V_7, V_9, V_{11}, V_{12}, V_{13}, V_{15} and V_{19}. All other fluxes can be expressed using the free fluxes ([S3]).

$$V_1 = 0.5*(V_3 + V_5 + V_9 + V_{19} - V_{12}) + V_2$$
$$V_2 = V_2$$
$$V_3 = V_3$$
$$V_4 = 0.5*(V_3 + V_5 + V_9 + V_{19} - V_{12})$$
$$V_5 = V_5$$
$$V_6 = V_7 + V_{12} - V_9 - V_{19}$$
$$V_7 = V_7$$
$$V_8 = V_{11} + V_{12} - V_9$$
$$V_9 = V_9$$
$$V_{10} = V_{19} + V_{11} \quad [S3]$$
$$V_{11} = V_{11}$$
$$V_{12} = V_{12}$$
$$V_{13} = V_{13}$$
$$V_{14} = V_{15} + V_{12} + V_{13} + V_{19}$$
$$V_{15} = V_{15}$$
$$V_{16} = V_{12} + V_{13}$$
$$V_{17} = V_{12} + V_{13} + 0.45*V_{19}$$
$$V_{18} = V_{12} + V_{13}$$
$$V_{19} = V_{19}$$
$$V_{out} = 0.5*(V_5 + V_9 + V_{19} - V_{12}) + V_2$$

Since the labeling patterns of all the input molecules are known (either unlabeled or fully labeled as tracer), the steady-state labeling patterns of all metabolites in the system can be calculated using the EMU

approach given any set of the nine fluxes. The calculated labeling patterns were compared to the measured ones. The best estimated flux set is obtained by minimizing the sum of squared residues (SSR) between the calculated and measured labeling patterns. The measured labeling patterns of six metabolites (glucose, glucose-6-phosphate, phosphoenolpyruvate, pyruvate, α-ketoglutarate and citrate) under two conditions ($^{13}C_3$ glycerol or $^{13}C_3$ lactate tracer) were used in this process. The numerical simulation of labeling patterns was achieved in R and the optimization was achieved with DEoptim package (54). 95% confidence intervals were calculated by (1) move one target flux away from the best-fit value by a small step (2) choosing a combination of the other fluxes that minimize the increase of SSR, (3) calculate the new SSR and repeat step (1) to (3) until the new SSR reached the cutoff for 95% confidence interval (55). The goodness of fit was tested by chi-square test, $X^2_{0.05}$ (df=49) = 66.34. The 49 degrees of freedom are based on 58 measurements (labeling fractions of six metabolites under two tracers) and having 9 unknown fluxes; 58 - 9 = 49.

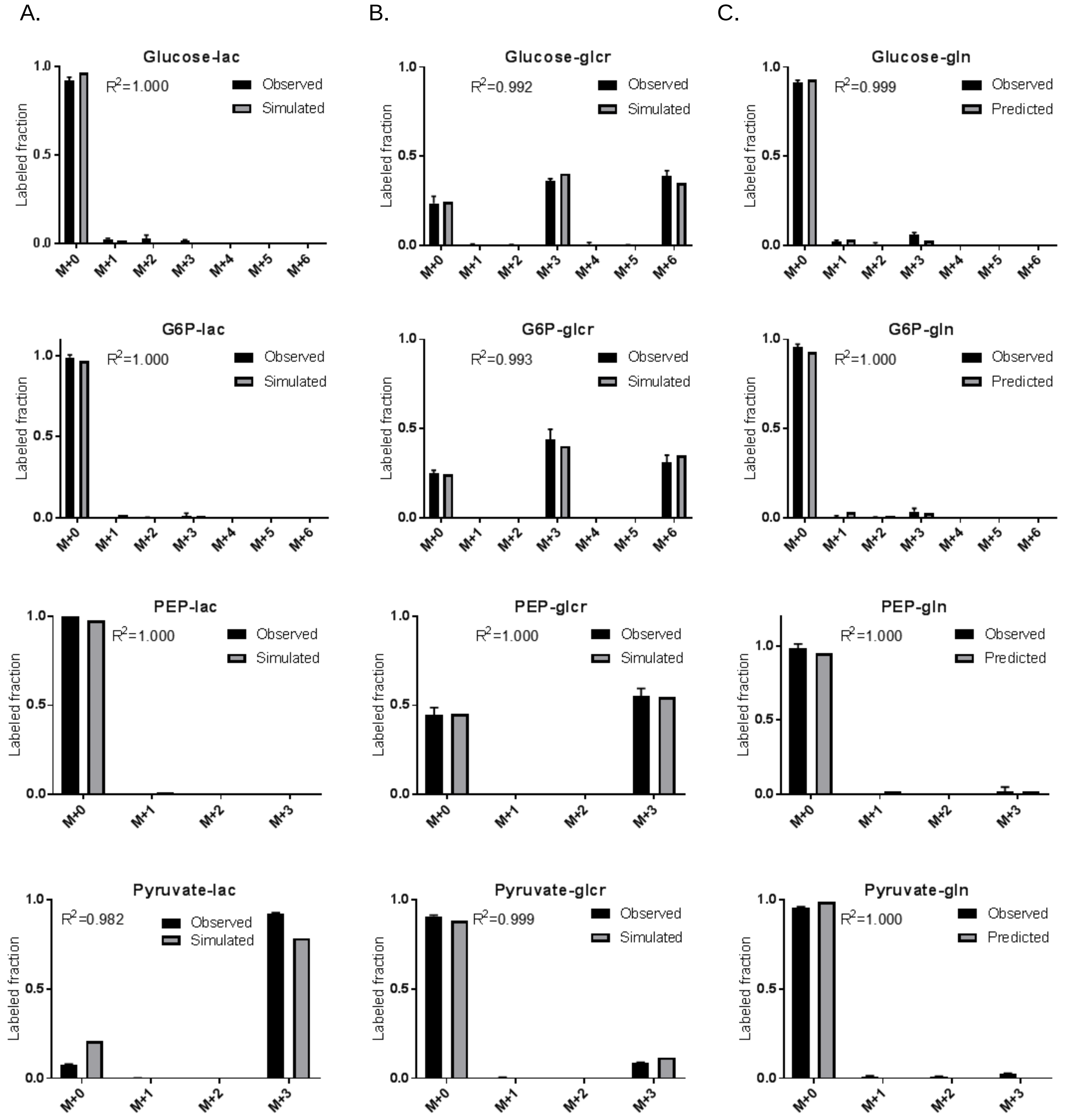

Figure S4: Labeling patterns of gluconeogenic intermediates from observation and simulation. **(a)** Observed and simulated labeling patterns of key metabolites in the $^{13}C_3$ lactate labeling experiment. **(b)** Observed and simulated labeling patterns of key metabolites in the $^{13}C_3$ glycerol labeling experiment. **(c)** Observed and simulated labeling patterns of key metabolites in the $^{13}C_5$ glutamine labeling experiment.
